# Associations between adolescents’ empathy and prosocial attributes before and during the COVID-19 pandemic

**DOI:** 10.1186/s12887-023-03977-4

**Published:** 2023-04-03

**Authors:** Xueqian Yang, Yirong He, Biru Luo, Li Zhao, Chuanya Huang, Shujuan Liao

**Affiliations:** 1 West China School of Nursing, Sichuan University/ Department of Nursing, West China Second University Hospital, Sichuan University, Chengdu, China; 2 Key Laboratory of Birth Defects and Related Diseases of Women and Children, Ministry of Education, West China Second University Hospital, Sichuan University, #No. 20, Section 3, People’s South Road, Wuhou District, Chengdu City, 610041 Sichuan Province People’s Republic of China; 3 Department of Nursing, West China Second University Hospital, Sichuan University/ West China School of Nursing, Sichuan University, Chengdu, China; 4 Department of Health Policy and Management, West China School of Public Health/ West China Forth Hospital, Sichuan University, Chengdu, 610041 China

**Keywords:** Adolescents, Empathy, Prosocial attributes, Longitudinal study

## Abstract

**Background:**

Adolescence is a formative period of social development. Adolescents have experienced considerable changes in their lives due to the COVID-19 pandemic. We conducted a longitudinal study to examine the effects of the COVID-19 pandemic on adolescents’ prosocial attributes and empathy, as well as their longitudinal bilateral relationships.

**Methods:**

A total of 2,510 students from five junior schools in Sichuan Province were recruited via random cluster sampling. Data were collected in December 2019 (Wave 1, before the outbreak of the pandemic) and July 2020 (Wave 2, during the pandemic) in Chengdu, Sichuan, China. Prosocial attributes and empathy were measured with the Positive Youth Development Scale (GPYDS) subscale and Chinese Empathy Scale, respectively.

**Results:**

During the pandemic, both empathy and prosocial attributes decreased significantly from 49.89 (9.12) and 49.89 (8.80) before to 48.29 (8.72) and 49.39 (9.26) (*p* < 0.001), respectively. A higher level of empathy at Wave 1 significantly predicted higher prosocial attributes at Wave 2 (β = 0.173, SE = 0.021, t = 8.430, *p* < 0.001). A lower prosocial attributes score predicted a significantly lower empathy score from Wave 1 to Wave 2 (β = 0.100, SE = 0.021, t = 4,884, *p* < 0.001).

**Conclusions:**

The COVID-19 pandemic has had detrimental effects on adolescents’ empathy and prosocial attributes. Special attention should be given to these two longitudinally associated factors in any social crisis, such as the COVID-19 pandemic, considering their importance for adolescents’ physical, mental, and social development.

## Background

Empathy, shaped by biological dispositions and caregiving experiences [1], is a core human social ability and the capacity to resonate with and reflect upon the feelings and mental states of others[2]. Empathy inspires adolescents to assist others and make prosocial contributions [3, 4], which is paramount for healthy adolescent development [5] and for promoting social interaction. Moreover, empathy can predict social competence in adulthood [6]. Evidence suggests that the changes in the empathy score were positively correlated with perceived social support from family, friends, and close people [5]. As adolescence is the second rapid period of development with major changes in the physical, psychological, and social environment, the development of empathy is quite important during this period.

Prosocial attributes indicate that humans have extraordinary altruism, such as social behaviour intended to benefit others, which sets humans apart from other animals [7] [8]. Both the understanding of others’ inner states (i.e., perspective taking) and the experience of feeling concern for others (i.e., empathy) are believed to facilitate prosocial behaviour. Much empirical research has consistently documented the beneficial role of prosocial behaviour both for the recipients’ well-being and the givers’ development [9].

Adolescence, the age period of approximately 13–18 years, is a developmental period marked by profound physical and psychological change [10]. There is a growing body of literature that recognizes adolescence as a critical period for the development of prosocial attributes and empathy [11]. Children increasingly think in terms of others as they enter early adolescence [11], which is exactly what we often call empathy. Adolescents’ involvement in empathy and pro-sociality not only promotes the growth of social and emotional self-regulation but also has long-term benefits, creating the necessary conditions for responsibility and citizenship throughout the lifespan. However, adolescence is also a sensitive period that could be easily influenced by external environments, such as a social crisis.

On March 11, 2020, the World Health Organization (WHO) declared that the outbreak of novel coronavirus disease (COVID-19) had become a pandemic [12]. As of December 28, 2021, there have been over 278 million confirmed cases of COVID-19, including 5.4 million deaths, reported to the WHO [13]. Many countries require people to isolate themselves at home or in a dedicated quarantine facility, and such social distancing strategies may have had multiple consequences on the lives of adolescents [14]. As schools closed, students began to study at home and experience restricted social activities and relationships [15]. Some rodent studies have shown that social isolation causes substantial changes in the brain and behaviour [16], especially if isolation occurs during development [17]. For adolescents, lockdown [16] may have resulted in a sudden social interruption [14]. Recent evidence suggests that adolescents showed more aggressive behaviour and lower levels of prosocial attributes, influenced by the COVID-19 pandemic [18, 19]. We speculate that the reduction in social contact with others may have affected the development of empathy and prosocial attributes of adolescents.

Large changes in the social environment (e.g., COVID-19) have played important roles in the development of empathy and prosocial attributes in adolescents[20]. Meanwhile, some studies reveal that there is a correlation between prosocial attributes and empathy among adolescents [21, 22]. However, most of the published studies were cross-sectional study designs [18, 23], with only one exception that attempted to investigate the associations between adolescents’ empathy and prosocial attributes before and during the COVID-19 pandemic [21]. Despite the longitudinal design of van de Groep, study [21], it was carried out in the Netherlands with a sample size of 58 adolescents, and different socioeconomic and cultural backgrounds might limit the generalization of the above-mentioned study to China. During the lockdown, China halted transportation in and out of the lockdown area and barred tens of millions of people from working or going to school, while Dutch participants [21] were allowed to go out for a walk or run [24]. In addition, different infection rates of COVID-19 0.154 in a Dutch study [25] and 0.006% [26] in China might have had different influences on the mental status of adolescents. Thus, we conducted a longitudinal study to assess the changes in adolescents’ empathy and prosocial attributes and explore their bilateral relationships before and during the COVID-19 pandemic in China, with the hypothesis that adolescents' empathy and prosocial attributes changed after the COVID-19 pandemic and there was also a correlation between empathy and prosocial attributes.

## Methods

### Participants and procedures

A total of 2,510 adolescents completed the whole study, and were recruited from five junior schools (one school in the Central district, two schools in the South district, and two schools in the North district) via random cluster sampling in Chengdu, Sichuan, China. Schools that met the following criteria were included in our study: located in Chengdu City, Sichuan Province; being public schools; having been founded for at least 6 years; and having a minimum number of more than 150 students. Because of the routine physical and mental assessment carried out by the included schools, underlying psychiatric or psychological disturbances did not exist in our sample. Data were collected in two waves: Wave 1 was carried out in December 2019 (before the COVID-19 pandemic), and Wave 2 was conducted in July 2020 (during the COVID-19 pandemic). Written informed consent was obtained from the school principals, parents, and students, respectively. Trained researchers offered explanations of the project, and participants were aware that they could withdraw at any stage of this study without any adverse consequences. After presenting their written informed consent, participants completed the questionnaires independently with necessary guidance, and the completed questionnaires were returned to researchers immediately. The whole session lasted around 30 min. Questionnaires with any missing data were excluded from the statistical analysis, because the sample size was sufficient to address our research questions. Finally, a total of 3,000 questionnaires were distributed and 2,510 questionnaires were collected with completed and qualified answers, with a response rate of 83.7%.

### Sample size

A minimum sample size of 425 was determined based on past research on empathy and prosocial attributes in adolescents [4]. Considering the 10% drop-out rate, the minimum sample size was 472 adolescents. To reduce sampling error [27], all adolescents who met the inclusion criteria were included in the study during the recruitment phase.

### Measurement tools

#### Demographics and COVID-19 infection history

Demographic information and COVID-19 infection history were measured with a self-developed data collection form containing the following five items: age (years), grade (4 to 9), gender (male and female), nationality (Han or minorities), and COVID-19 infection history (Did you or your family members contract COVID-19?).

#### Prosocial Attributes

Prosocial attributes were measured using the 80-item Positive Youth Development Scale (GPYDS) subscale. It is an indigenous Chinese measure of PYD that has been validated among Chinese adolescents [28–31]. The prosocial attributes subscale contains 10 items and covers two subscales, “prosocial norms” (PN) and “prosocial involvement” (PI), which assess young people's beliefs, behaviour guidelines, participation in prosocial behaviours and maintenance of prosocial norms. One item of the PN subscale is “I care about the unfortunate people in society”, and a sample item of the PI subscale is “I will try my best to contribute to the school or society”. Participants indicated how they felt on a 6-point scale, with 1 indicating “strongly disagree”, and 6 indicating “strongly agree”. The prosocial attributes subscale has adequate internal consistency and validity, with a Cronbach's alpha coefficient of 0.90 [30].

#### Empathy

The Chinese Empathy Scale [32] was employed in this study. Eleven items were answered on a 6-point Likert scale, ranging from 1 (“strongly disagree”) to 6 (“strongly agree”), with higher ratings indicating greater empathic concern. A sample item reads “Understanding of the situation of others and emotional resonance”. The scale was found to be internally consistent [32](α = 0.74; mean inter-item correlation = 0.21; mean item-total correlation = 0.39). According to our data, the Cronbach's alpha coefficients of the empathy scale at Time 1 and Time 2 were 0.80 and 0.78, respectively.

### Statistical methods

Statistical analyses were conducted using SPSS 26.0 and MPlus 8.0. For descriptive statistics, data following a Gaussian distribution were expressed by the mean and standard deviation, and medians and interquartile ranges were presented when the data were nonnormally distributed. Due to the nonnormal distribution of prosocial attributes and empathy, the Wilcoxon signed-rank test (using SPSS software, version 26.0) was adopted to calculate their variations before and during the COVID-19 pandemic. The cross-lagged analysis (using Mplus software, version 8.0) was adopted to explore the longitudinal bilateral associations between them. All statistical tests used were two-sided, and a *P* value of less than 0.05 was considered statistically significant.

## Results

### Basic information of participants

A total of 2,510 participants, 1,229 females (48.96%) and 1,281 males (51.04%), with a mean age of 14.2 years (SD = 0.9 years, range: 13–18 years), participated in this longitudinal study. The majority of participants were in grades 7–9. Among them, 2,487 (99.08%) were Han majority, and 23 (0.92%) were members of minority ethnicities. Eighty-four participants reported COVID-19 infection history. Detailed information is shown in Table 1.

**Table 1:** Descriptive statistics (*N* = 2510)

Variables	Mean ± SD / n (%)
Age	14.2 ± 0.9
Gender	
Male	1281 (51.04)
Female	1229 (48.96)
Grade	
4 ~ 6	84 (3.35)
7 ~ 9	2426 (96.65)
Nationality	
Han	2487 (99.08)
Minorities	23 (0.92)
COVID-19 infection history	
Yes	84 (3.35)
No	2426 (96.65)

### Variations in prosocial attributes and empathy before and during the COVID-19 pandemic

The Wilcoxon signed-rank test was used to compare prosocial attributes and empathy before (Wave 1) and during (Wave 2) the COVID-19 pandemic. Participants’ empathy decreased from 48.85 before to 48.29 during the pandemic (*p* < 0.001), with differences in both males (*p* = 0.010) and females (*p* = 0.005). The same descending trend was found in adolescents’ prosocial attributes as well (*p *= 0.030), while no difference was confirmed when gender and COVID-19 infection history were analysed as stratifying factors. Table 2 summarizes the differences in prosocial attributes and empathy in adolescents before and during the COVID-19 pandemic.

**Table 2:** Differences in prosocial attributes and empathy among adolescents at the two waves (*N* = 2510)

Variables	Before M (P25, P75)	During M (P25, P75)	Z	*p* value	95%CI
Prosocial Attributes					
Total	49.89 (45,57)	49.39 (44,57)	-2.165	.030	0.111,0.879
Males	49.25 (43,57)	48.76 (45,57)	-1.204	.229	-0.112,1.084
Females	50.56 (46,57)	50.06 (45,57)	-1.879	.060	0.028,0.981
Empathy					
Total	48.85 (43,55)	48.29 (42,54)	-3.760	.000	0.188,0.926
Males	47.71 (42,54)	47.19 (41,53)	-2.570	.010	-0.034,1.077
Females	50.03 (44,56)	49.44 (44,55)	-2.779	.005	0.110,1.078

### Longitudinal bilateral associations between prosocial attributes and empathy

**Fig. 1:**
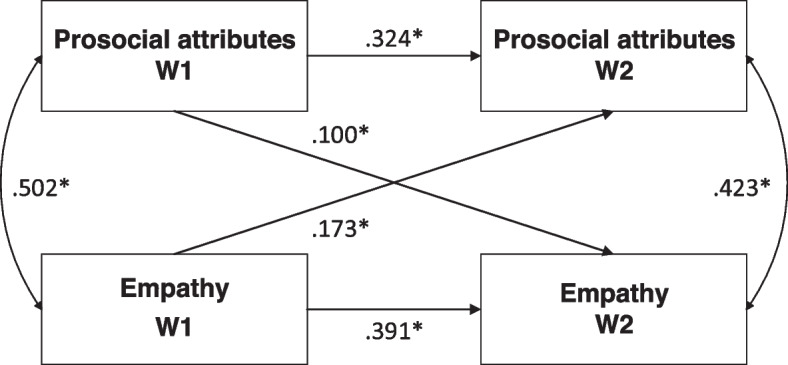
Cross-lagged model between prosocial attributes and empathy. Note: The two-way arrow in the chart indicates the result of correlation analysis, with the data of the correlation coefficient; the one-way arrow indicates the result of path analysis, with the data of the standardized regression coefficient (β). *: *p* < 0.001. Prosocial attributes W1: prosocial attributes score before the COVID-19 pandemic; Prosocial attributes score W2: prosocial attributes score during the COVID-19 pandemic; Empathy W1: empathy score before the COVID-19 pandemic; Empathy W2: empathy score during the COVID-19 pandemic

We conducted cross-lagged analyses in Mplus 8.0 (Muthén & Muthén, 1998–2012) using maximum likelihood (ML) to explore the longitudinal associations between prosocial attributes and empathy. Longitudinal bilateral relations between empathy and prosocial attributes were found in our model. Prosocial attributes in Wave 1 were significantly associated with empathy at Wave 2 (β = 0.100, SE = 0.021, t = 4.884, *p* < 0.001) (Fig. 1). Higher empathy at Wave 1 significantly predicted higher prosocial attributes at Wave 2 (β = 0.173, SE = 0.021, t = 8.430, *p* < 0.001). The cross-lagged model presented other structural paths as well: empathy at Wave 1 was positively correlated with empathy at Wave 2 (β = 0.391, SE = 0.019, t = 20.14, *p* < 0.001), and the same correlation was found in prosocial attributes as well (β = 0.342, SE = 0.020, t = 16.22, *p* < 0.001). Empathy was positively associated with prosocial attributes both before and during the COVID-19 pandemic, with correlation coefficients of 0.502 (SE = 0.015, t = 33.591, *p* < 0.001) and 0.423 (SE = 0.016, t = 25.786, *p* < 0.001), respectively.

## Discussion

In the current study, we investigated not only the effects of the COVID-19 pandemic on Chinese adolescents’ prosocial attributes and empathy, but also the longitudinal associations between prosocial attributes and empathy before and during the pandemic. Our findings contributed largely to the known literature, which were almost all cross-sectional studies.

One unanticipated finding related to the first part of the study is that both the prosocial attribute score and the empathy score decreased significantly during the COVID-19 pandemic lockdown. Ezpeleta, L. et al*.* [33] also found that lower prosocial attributes were mostly associated with reduced activity, excessive parental discipline and psychological control, and lack of emotional expression. Our study reached this conclusion with a larger sample size. Prior studies have already shown that empathy comes from emotional interactions with others and self-efficacy projects and experiences in daily life [34]. Due to the COVID-19 pandemic, adolescents were isolated at home and had reduced contact with others. Limited physical and emotional interactions with others might be the reason for the decline in prosocial attributes and empathy among adolescents [35].

According to our findings, empathy predicted prosocial attributes scores concurrently and longitudinally. These findings are generally in line with the existing literature reporting that empathy is positively associated with adolescent prosocial attributes [3, 4, 36]. Adolescents with greater prosocial attributes tended to report greater empathy. Adolescents with a lower level of empathy before the COVID-19 pandemic were more likely to report a lower level of empathy during the pandemic. For example, a recent study demonstrated the detrimental effects of the COVID-19 pandemic on adolescents’ empathy and opportunities for prosocial actions [21].The results of another study [37] showed an increase in the proportion of adolescents with a low level of prosocial tendency. However, a cross-sectional study reported a higher level of empathy and more generous feelings towards others in the context of the COVID-19 pandemic [38]. The differences in sociodemographic backgrounds might be the reason for the inconsistencies in findings. The majority of the subjects in BEAMES J R's study [38] were female (72%), while only 48.96% of our study subjects were female. The gender difference may have contributed to the higher level of empathy obtained in their study. Another reason for this inconsistency might be the differences in data collection and study design. In BEAMES J R's study [38], retrospective data collection was used, while our study assessed the outcomes prospectively. The cross-sectional study design of BEAMES J R's study [38] might also result in differences in findings from our longitudinal design.

Our findings may be somewhat impacted by the increased access to electronic devices during the lockdown period [39, 40]. It is likely to increase the exposure of adolescents to adverse information and violent games [40], which was strongly suggested as a causal risk factor for decreased empathy and prosocial behaviour [41]. A study has shown that social interactions and the promotion of positive emotions predisposed adolescents to act prosocially [42], and social interactions offer better opportunities to emerge from crises. Meanwhile, our findings highlight empathy and prosocial attributes of adolescents experiencing social crises such as the COVID-19 pandemic and offer policymakers and health professionals who will possibly benefit from utilizing the features of adolescents while making health-related policies and decisions. Mental preparedness should be highlighted to mitigate possible public emergencies in the future. Thus, to develop a full picture of the impact of the COVID-19 pandemic, additional studies will be needed to discover the long-term consequences of the pandemic in reducing prosocial attributes and empathy in adolescents. Future longitudinal research to follow the trajectories of adolescents’ prosocial attributes and empathy after the pandemic is needed.

Despite the large sample size and the longitudinal design of this study, there were several limitations. First, this study was conducted only in Sichuan Province, which may limit the representativeness of our sample. Further multicentre work with a larger sample size is needed. Second, the full impact of the COVID-19 pandemic will take time, while the time range between Wave 1 and Wave 2 in our study was slightly short. Thus, a longer follow-up is needed to illustrate this. Third, it will be more convincing if we assessed the time spent using electronic devices, and this would be considered in our further studies.

## Conclusions

COVID-19 had detrimental effects on adolescents' empathy and prosocial attributes. Longitudinal bilateral relationships between prosocial attributes and empathy were also discovered. Special attention should be given to the prosocial attributes and empathy of adolescents experiencing social crises such as the COVID-19 pandemic, considering their importance for the physical, mental and social development of adolescents. Our findings might offer valuable data for policymakers and health professionals who will possibly benefit from utilizing these finding about adolescents’ characteristics while making health-related policies and decisions. Future multicentred work with larger sample sizes and longer follow-ups will be needed to fully illustrate the long-term impact of the COVID-19 pandemic on the prosocial attributes and empathy of adolescents.

## Data Availability

The datasets used and/or analysed during the current study are available from the corresponding author on reasonable request.

## References

[1] Levy J, Goldstein A, Feldman R (2019). The neural development of empathy is sensitive to caregiving and early trauma. Nat Commun.

[2] Spinrad TL, Eisenberg N, Cumberland A, Fabes RA, Valiente C, Shepard SA, Reiser M, Losoya SH, Guthrie IK (2006). Relation of emotion-related regulation to children's social competence: a longitudinal study. Emotion (Washington, DC).

[3] Carlo G, Padilla-Walker LM, Nielson MG (2015). Longitudinal bidirectional relations between adolescents’ sympathy and prosocial behavior. Dev Psychol.

[4] Van der Graaff J, Carlo G, Crocetti E, Koot HM, Branje S (2018). Prosocial behavior in adolescence: gender differences in development and links with empathy. J Youth Adolesc.

[5] Shima T, Nakao H, Tai K, Shimofure T, Jesmin S, Arai Y, Kiyama K, Onizawa Y. The influences of changes in physical activity levels with easing restriction of access to the University Campus on empathy and social supports in College Students During the COVID-19 pandemic. Asia-Pac J Public Health. 2022;34(4):406–10. 10.1177/10105395221083381 35249364

[6] Allemand M, Steiger AE, Fend HA (2015). Empathy development in adolescence predicts social competencies in adulthood. J Pers.

[7] Fehr E, Fischbacher U (2003). The nature of human altruism. Nature.

[8] Damon W, Lerner RM, Eisenberg N. Handbook of child psychology, social, emotional, and personality development, Vol. 3, 6th ed. Hoboken: Wiley; 2006.

[9] Weinstein N, Ryan RM (2010). When helping helps: autonomous motivation for prosocial behavior and its influence on well-being for the helper and recipient. J Pers Soc Psychol.

[10] Kral TRA, Stodola DE, Birn RM, Mumford JA, Solis E, Flook L, Patsenko EG, Anderson CG, Steinkuehler C, Davidson RJ (2018). Neural correlates of video game empathy training in adolescents: a randomized trial. NPJ Sci Learn.

[11] van den Bos W, Westenberg M, van Dijk E, Crone EA (2010). Development of trust and reciprocity in adolescence. Cogn Dev.

[12] Coronavirus disease 2019 (COVID-19) situation report – 62. 2020.

[13] Weekly epidemiological update on COVID-19 - 28 December 2021 [https://www.who.int/publications/m/item/weekly-epidemiological-update-on-covid-19---28-december-2021]

[14] Brooks SK, Webster RK, Smith LE, Woodland L, Wessely S, Greenberg N, Rubin GJ (2020). The psychological impact of quarantine and how to reduce it: rapid review of the evidence. Lancet.

[15] Oosterhoff B, Palmer CA, Wilson J, Shook N (2020). Adolescents' motivations to engage in social distancing during the COVID-19 Pandemic: Associations With Mental and Social Health. J Adolesc Health.

[16] Matthews GA, Tye KM (2019). Neural mechanisms of social homeostasis. Ann N Y Acad Sci.

[17] Burke AR, McCormick CM, Pellis SM, Lukkes JL (2017). Impact of adolescent social experiences on behavior and neural circuits implicated in mental illnesses. Neurosci Biobehav Rev.

[18] Wang L, Zhang Y, Chen L, Wang J, Jia F, Li F, Froehlich TE, Hou Y, Hao Y, Shi Y (2021). Psychosocial and behavioral problems of children and adolescents in the early stage of reopening schools after the COVID-19 pandemic: a national cross-sectional study in China. Transl Psychiatry.

[19] Wiguna T, Anindyajati G, Kaligis F, Ismail RI, Minayati K, Hanafi E, Murtani BJ, Wigantara NA, Putra AA, Pradana K (2020). Brief research report on adolescent mental well-being and school closures during the COVID-19 Pandemic in Indonesia. Front Psych.

[20] Silke C, Brady B, Boylan C, Dolan P (2018). Factors influencing the development of empathy and pro-social behaviour among adolescents: a systematic review. Child Youth Serv Rev.

[21] van de Groep S, Zanolie K, Green KH, Sweijen SW, Crone EA (2020). A daily diary study on adolescents' mood, empathy, and prosocial behavior during the COVID-19 pandemic. PLoS One.

[22] Rieffe C, Camodeca M (2016). Empathy in adolescence: relations with emotion awareness and social roles. Br J Dev Psychol.

[23] Galang CM, Johnson D, Obhi SS (2021). Exploring the relationship between empathy, self-construal style, and self-reported social distancing tendencies during the COVID-19 pandemic. Front Psychol.

[24] Poelman MP, Gillebaart M, Schlinkert C, Dijkstra SC, Derksen E, Mensink F, Hermans RCJ, Aardening P, de Ridder D, de Vet E (2021). Eating behavior and food purchases during the COVID-19 lockdown: a cross-sectional study among adults in the Netherlands. Appetite.

[25] worldometers/coronavirus/country/netherlands/ [https://www.worldometers.info/coronavirus/country/netherlands/]

[26] 截至7月23日24时新型冠状病毒肺炎疫情最新情况 [http://www.gov.cn/xinwen/2020-07/24/content_5529679.htm]

[27] Sedgwick P (2012). What is sampling error?. BMJ.

[28] Shek DTL, Siu AMH, Tak Yan L (2016). The Chinese Positive Youth Development Scale. Res Soc Work Pract.

[29] Shek DTL, Ma CMS (2009). Dimensionality of the Chinese Positive Youth DEVELOPMENT Scale: confirmatory factor analyses. Soc Indic Res.

[30] Zhou Z, Shek DTL, Zhu X (2020). The importance of positive youth development attributes to life satisfaction and hopelessness in mainland Chinese adolescents. Front Psychol.

[31] Shek DTL, Zhao L, Dou D, Zhu X, Xiao C (2021). The impact of positive youth development attributes on posttraumatic stress disorder symptoms among Chinese Adolescents under COVID-19. J Adolesc Health.

[32] Shek DTL, Ma CMS, Lin L. The Chinese Adolescent Materialism Scale: psychometric properties and normative profiles. Int J Disabil Hum Dev. 2014;13(2):285–95.

[33] Ezpeleta L, Navarro JB, de la Osa N, Trepat E, Penelo E. Life conditions during COVID-19 lockdown and mental health in spanish adolescents. Int J Environ Res Public Health. 2020;17(19):7327. 10.3390/ijerph17197327 PMC7579639 33036461

[34] Richards R, McGee R, Williams SM, Welch D, Hancox RJ (2010). Adolescent screen time and attachment to parents and peers. Arch Pediatr Adolesc Med.

[35] Ma X, Wang X (2021). The role of empathy in the mechanism linking parental psychological control to emotional reactivities to COVID-19 pandemic: a pilot study among Chinese emerging adults. Personal Individ Differ.

[36] Carrizales A, Branje S, Lannegrand L (2021). Disentangling between- and within-person associations between empathy and prosocial behaviours during early adolescence. J Adolesc.

[37] Hu Y, Qian Y (2021). COVID-19 and Adolescent Mental Health in the United Kingdom. J Adolescent Health.

[38] Beames JR, Li SH, Newby JM, Maston K, Christensen H, Werner-Seidler A (2021). The upside: coping and psychological resilience in Australian adolescents during the COVID-19 pandemic. Child Adolesc Psychiatry Ment Health.

[39] Mohan A, Sen P, Shah C, Jain E, Jain S (2021). Prevalence and risk factor assessment of digital eye strain among children using online e-learning during the COVID-19 pandemic: digital eye strain among kids (DESK study-1). Indian J Ophthalmol.

[40] Siste K, Hanafi E, Sen LT, Murtani BJ, Christian H, Limawan AP, Siswidiani LP (2021). Adrian: implications of COVID-19 and lockdown on internet addiction among adolescents: data from a developing country. Front Psych.

[41] Anderson CA, Shibuya A, Ihori N, Swing EL, Bushman BJ, Sakamoto A, Rothstein HR, Saleem M. Violent video game effects on aggression, empathy, and prosocial behavior in eastern and western countries: a meta analytic review. Psychol Bull. 2010;136(2):151-73. 10.1037/a0018251 20192553

[42] Mesurado B, Resett S, Tezón M, Vanney CE (2021). Do positive emotions make you more prosocial? direct and indirect effects of an intervention program on prosociality in colombian adolescents during social isolation due to COVID-19. Front Psychol.

